# Human Activities and Climate Change Accelerate the Spread Risk of *Hyphantria cunea* in China

**DOI:** 10.3390/insects17020154

**Published:** 2026-01-30

**Authors:** Mu Duan, Jing Ning, Gejiao Wang, Zhaochen Xu, Shengming Li, Zhen Zhang, Longwa Zhang, Lilin Zhao

**Affiliations:** 1 Anhui Provincial Key Laboratory of Forest Resources and Silviculture, School of Forestry & Landscape Architecture, Anhui Agricultural University, Hefei 230036, China; duanmu99cn@163.com; 2 State Key Laboratory of Animal Biodiversity Conservation and Integrated Pest Management, Institute of Zoology, Chinese Academy of Sciences, Beijing 100101, China; ningjing@ioz.ac.cn (J.N.); wgj1124@outlook.com (G.W.); zhenz1617@gmail.com (Z.Z.); 3 Shanxi Fen River Plain Farmland Shelterbelt Ecosystem National Observation Research Station, Jinzhong 030801, China; 4 School of Biological and Behavioral Sciences, Queen Mary University of London, London E1 4NS, UK; 5 College of Life Science, Hebei University, Baoding 071002, China; chenney0312@163.com (Z.X.); 20238017117@stumail.hbu.edu.cn (S.L.)

**Keywords:** *Hyphantria cunea*, species distribution models, human activity, climate change, habitat suitability prediction

## Abstract

Anthropogenic activities and climate change have accelerated biological invasions, leading to profound ecological, economic, social, and health impacts. The invasive species fall webworm (*Hyphantria cunea*) has been reported to have outbreaks in areas with climate anomalies and human settlements in recent years, highlighting the necessity to explore the species’ suitable habitat and associated future changes. We built an ensemble species distribution model using Random Forest, MaxEnt, and Support Vector Machine, achieving excellent predictive performance (AUC = 0.996). Our results identify human settlement density as the dominant driving factor, with a contribution > 50%, far exceeding climatic and forest structure variables. Therefore, densely urbanized regions such as Beijing–Tianjin–Hebei, the Liaodong Peninsula, and the North China Plain comprise the current highly suitable areas. Future climate projections suggest a continued expansion of the suitable habitat for *H. cunea*, with the most pronounced growth expected under the high-emission pathway (SSP5-8.5), where human activity is greatest. Such a correlation indicates that highly urbanized regions should be given priority for corresponding monitoring and control measures. As climate warming continues, northeastern China will face escalating invasion risks. Conversely, some regions within the Yangtze River Delta may become less suitable for the habitation of *H. cunea*. These findings provide insightful guidance for region-specific surveillance, quarantine measures, and the precision management of *H. cunea* in China.

## Simple Summary

Invasive pests are spreading more rapidly worldwide as a result of a warming climate and increasing human activities, with particularly severe impacts on forest ecosystems. The fall webworm is an important quarantine pest in China, as this leaf-feeding moth can cause serious damage to forests, orchards, and urban trees. This study aims to identify the areas where fall webworms are most likely to occur at present, and to examine potential future changes in these risk ranges. By combining historical outbreak records with regional information on climate conditions, forest distribution, and human settlements, we developed a predictive framework to map areas at risk across China. The results demonstrate that densely populated regions—such as cities and their surrounding areas—are far more conducive to the habitation of fall webworms than sparsely populated areas. At present, areas at highest risk are primarily concentrated in the Beijing–Tianjin–Hebei region and the North China Plain. In the future, strong climate-warming scenarios are projected to induce further expansion of the habitable areas for fall webworms. These findings highlight the pivotal role of human activities in driving pest dispersal and provide practical guidance for early warning, targeted monitoring, and effective forest protection.

## 1. Introduction

Forests are one of the core components of the global terrestrial ecosystem, undertaking key functions such as climate regulation, biodiversity maintenance, soil conservation, and carbon storage [1]. According to the latest assessment by the Food and Agriculture Organization (FAO), global forest area in 2025 is estimated at 4.14 billion hectares (accounting for 32% of the total land area), serving as the primary global terrestrial carbon sink [2]. Simultaneously, forest habitats harbor roughly 80% of terrestrial biodiversity [3], supporting food security, livelihoods, and renewable biomaterial and energy supplies for billions of people worldwide [2]. Hence, maintaining forest health and functional integrity is crucial to climate stability and human welfare.

However, forests are facing multidimensional pressures. Global changes such as a warming climate, more frequent extreme weather events, and land-use change, combined with intensive human interventions (e.g., international trade, tourism), have significantly increased ecosystem vulnerability. In this context, invasions by exotic pests have become a major driving force of forest degradation, functional losses, and severe economic damage. These invasion events are not isolated occurrences, but rather symptoms of Anthropocene ecological disruptions—global trade networks provide “highways” for species to cross geographic barriers, while climate change brings about desirable conditions for invasive species, enabling them to settle in and colonize new territories [4,5,6]. Among the various invasive organisms, forest insects are particularly notable for their powerful dispersal and destructive capabilities [7]. The economic loss caused by alien species worldwide has reached the scale of hundreds of billions of dollars [8,9,10], with forest insect invasions contributing significantly [11].

The fall webworm (*Hyphantria cunea*), of the order Lepidoptera and family Erebidae, is a cosmopolitan quarantine pest with extraordinarily strong invasive potential. Native to North America and first detected in China in Dandong, Liaoning Province, in 1979, it has since rapidly spread across northeastern, northern, and parts of central China. According to the 2025 infested area announcement, the fall webworm has spread to 13 provinces and 90 cities (596 cities, counties, and districts) in China [12]. Owing to the voracious feeding habits of its larvae, the species can utilize more than 600 host plants, including many cultivated trees and shrubs. During outbreak periods, its extensive leaf consumption causes the severe defoliation of host plants, weakens photosynthetic capacity, and reduces vegetative productivity [13,14]. Therefore, assessing its potential expansion trends, risk areas, and driving factors in the context of human activities and global climate change is critical for understanding the invasion dynamics of exotic pests, as well as for improving national biosecurity early-warning systems and developing adaptive management strategies [15].

In this study, we aimed to (1) model the current potential suitable habitat of *H. cunea* in China using an ensemble species distribution modeling (SDM) framework, (2) quantify the relative contributions of climatic and human activity variables to its spatial distribution, and (3) project potential changes in suitable habitat under future climate scenarios. In addition, we integrated SDM outputs with vegetation carbon density data to provide a primary assessment of the potential exposure of forest carbon stocks to fall webworm invasion. By explicitly linking invasion risk with forest carbon distribution, this study directly addresses the increasing concern over forest insect invasions under global change and offers spatially explicit scientific evidence to support targeted monitoring, early warning, and region-specific prevention and management strategies for *H. cunea* in China.

## 2. Materials and Methods

### 2.1. Data Sources

Previous occurrence data of fall webworm invasion in China were obtained from the National Forestry and Grassland Administration [16] and the Global Biodiversity Information Facility (https://doi.org/10.15468/dl.rz82rb, accessed on 19 June 2023) [17]. A total of 635 occurrence records were collected from databases, published literature, official reports, and monitoring programs (Table S1). All occurrence records were examined, and those with the following geographical characteristics were removed: location within the national capital, provincial centers, research institutions, museums, and the ocean; duplicates with identical latitude and longitude; or coordinates (0,0). Next, a stratified random thinning algorithm implemented in the R package (version 4.3.2), spThin (100 replicates), was applied with a minimum distance of 50 km between points to effectively reduce geographic sampling bias [18]. After organization and filtering, a total of 174 occurrence points of fall webworm were used for subsequent analyses.

### 2.2. Selection of Environmental Variables

Bioclimatic variables were obtained from WorldClim (http://www.worldclim.org/, accessed on 1 July 2025). The WorldClim dataset provides bioclimatic data for 1970–2000 as well as climate projection data under different socioeconomic scenarios for every 20 years from 2021 to 2100 (i.e., 2021–2040, 2041–2060, 2061–2080, and 2081–2100). We also incorporated climate model projections from the Shared Socioeconomic Pathways (SSP) under the IPCC Sixth Assessment Coupled Model Intercomparison Project to account for future climatic changes. Specifically, greenhouse gas emission scenarios SSP1-2.6, SSP2-4.5, SSP3-7.0, and SSP5-8.5 represent low, moderate, moderate-to-high, and high forcing [19]. All four scenarios were selected to predict the potential spatial distribution of fall webworm in China. To enhance data authenticity, we also considered three commonly used human activity impact indices and five forest distribution factors: human settlement distribution, road density, nighttime light index, forest integrity, forest density, natural forest cover, plantation forest cover, and urban forest index. All of the above raster data were processed to a spatial resolution of 2.5 arcminutes to ensure spatial consistency among the indices.

**Figure A1:**
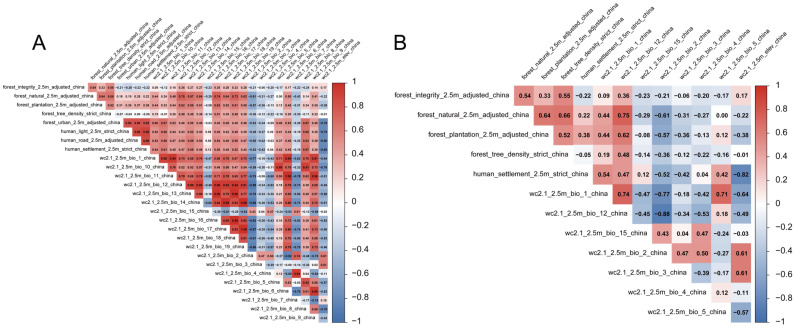
Spearman correlation matrices of environmental variables: (**A**) correlations among all candidate variables; (**B**) correlations among variables retained after collinearity screening (|r| < 0.8).

Spearman correlation coefficients were used to screen the variables and overcome multicollinearity among the nineteen climate factors, as well as the human and forest indices. Climate factors with a correlation coefficient > 0.8 were considered highly correlated and were therefore excluded [20]. Finally, thirteen environmental variables that were deemed significantly influential on the ecology and distribution of *H. cunea* were selected: forest integrity, natural forest cover, plantation forest cover, forest density, human settlement density, annual mean temperature (Bio1), mean diurnal temperature range (Bio2), isothermality (Bio3), temperature seasonality (Bio4), maximum temperature of the warmest month (Bio5), annual precipitation (Bio12), precipitation seasonality (Bio15), and elevation (Figure A1B, Appendix A).

### 2.3. Species Distribution Modeling

Occurrence data with climate and human activity variables were used to generate pseudo-absence points using a random sampling approach. Specifically, 200 pseudo-absence points were randomly generated within the national boundary of China. The R (version 4.3.2) package SDM [21] was then used to implement ensemble SDMs, predicting species distribution via a high-performance computing cluster. Next, 75% of the occurrence data for *H. cunea* (presence–absence) were randomly selected as training data, and the remaining 25% were used for evaluation. This subsampling process was repeated 10 times to account for uncertainty associated with data partitioning [22].

Fourteen commonly used high-performance SDM algorithms were employed in the ensemble model: Random Forests [23], Multivariate Adaptive Regression Splines (MARS) [24], Support Vector Machine, MaxEnt (maximum entropy) [25], Recursive Partitioning and Regression Trees (RPART), Maxlike, Generalized Additive Model (GAM) [26], Generalized Linear Model (GLM), Polynomial Generalized Linear Model (GLM-POLY), DOMAIN (Environmental Similarity Model) [27,28], BIOCLIM (bioclimatic envelope model), GLMNET (regularized GLM) [23], Mahalanobis Distance Model, and maxNet (an implementation of MaxEnt).

We then performed a 10-fold bootstrap ROC curve analysis for the fourteen species distribution models. True Skill Statistic (TSS) [29] and the area under the ROC curve (AUC) were used to calibrate and validate the robustness of the fourteen evaluated models (model selection) [22]. The values range from 0.5 to 1, with higher values indicating better predictive accuracy [25]. We selected the three models with TSS > 0.95, AUC > 0.996, and ΔAUC < 0.01 to form an ensemble model for predicting species distribution, and validated it again using ROC analysis.

Next, we applied a permutation-based variable importance evaluation to quantify the relative contribution of each environmental and human activity variable to predicting a suitable habitat for *H. cunea*. This method involves randomly shuffling the values of one variable at a time after model training and assigning an importance metric to that variable by calculating the decrease in model predictive performance. After computation, response curves for the key variables were generated to assess how variations in each variable affect model outcomes.

The map of the potentially suitable habitats for *H. cunea* under current climate conditions has values ranging from 0 to 1. These values were reclassified into five habitat categories: “highly suitable” (0.8–1), “moderately high suitable” (0.6–0.8), “moderately suitable” (0.4–0.6), “marginally suitable” (0.2–0.4), and “unsuitable” (<0.2) [25]. After modeling the spatial range of suitable habitat using current climate data, predictions were made for sixteen climate-time scenarios (four future climate scenarios, SSP1-2.6, SSP2-4.5, SSP3-7.0, SSP5-8.5, each at four future periods: 2021–2040, 2041–2060, 2061–2080, and 2081–2100) to forecast the extent of suitable habitat.

### 2.4. Estimation of Potential Carbon Stock

To assess the potential carbon stock that may be threatened by the invasion of the fall webworm (*Hyphantria cunea*), we conducted a first-order spatial estimation by integrating predicted suitable habitat areas with literature-based vegetation carbon density data. Predicted suitable habitats under current and future climate scenarios were derived from the ensemble species distribution models described above. A binary classification based on the optimal threshold value was used to identify suitable areas, and total suitable habitat areas were calculated for each climate scenario and period.

The national average carbon density of China in 2025, obtained from the FAO, was used as a representation for vegetation carbon storage capacity [2]. This value reflects current carbon storage conditions at the national scale and is consistent with carbon accounting approaches adopted in large-scale assessments of terrestrial carbon stocks and carbon sink vulnerability [1,30].

## 3. Results

### 3.1. Historical Spread and Outbreak Dynamics of Hyphantria cunea in China

**Figure 1:**
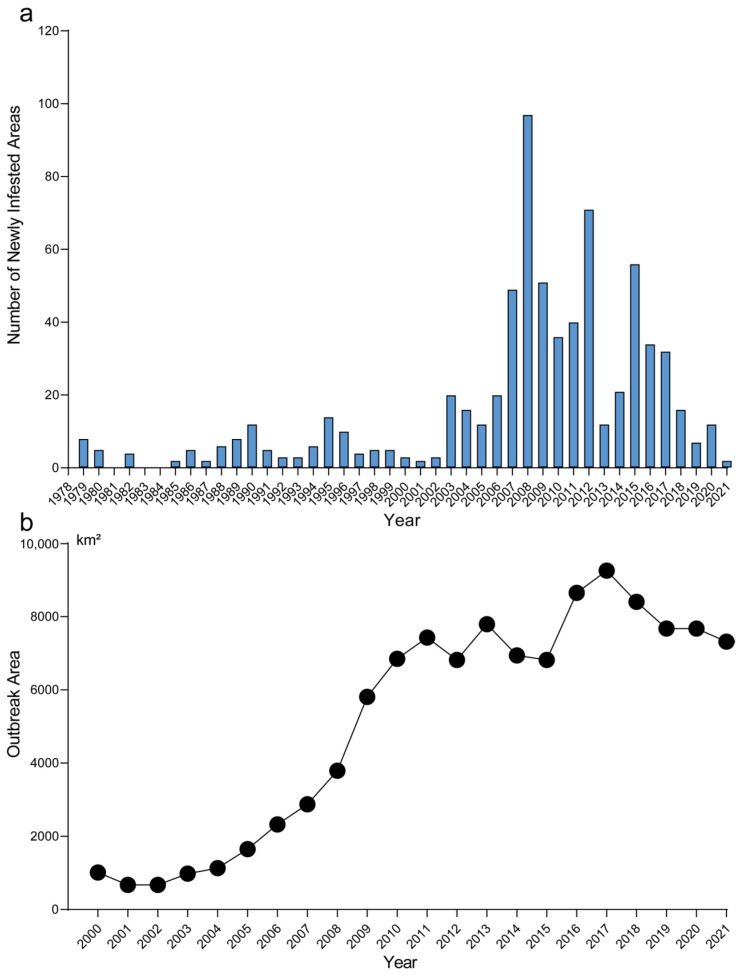
The invasion history of fall webworm in China. (**a**) Annual number of newly infested county-level administrative units of fall webworm in China from 1979 to 2021. (**b**) Annual outbreak area of fall webworm in China from 2000 to 2021.

First discovered in Liaoning Province in 1979, the spread of *H. cunea* in China can be described in three stages. Before 2002, its spread was slow and steady, with an annual increase of 4.4 newly infested areas. From 2002 to 2018, the spread became more rapid, averaging 37 newly infested areas per year; after 2018, the spread slowed down (Figure 1a). In terms of area experiencing outbreaks (Figure 1b), there was an exponential increase from 2000 to 2011. The growth peaked in 2018 and then declined each year thereafter. These results indicate that although the spread of fall webworm has decelerated, the possibility of potential outbreaks remains, particularly in those regions where the expansion of suitable areas may occur.

### 3.2. Comparison of Human Activity, Forest Type, and Climate Change on the Habitat Suitability of Fall Webworm

**Figure 2:**
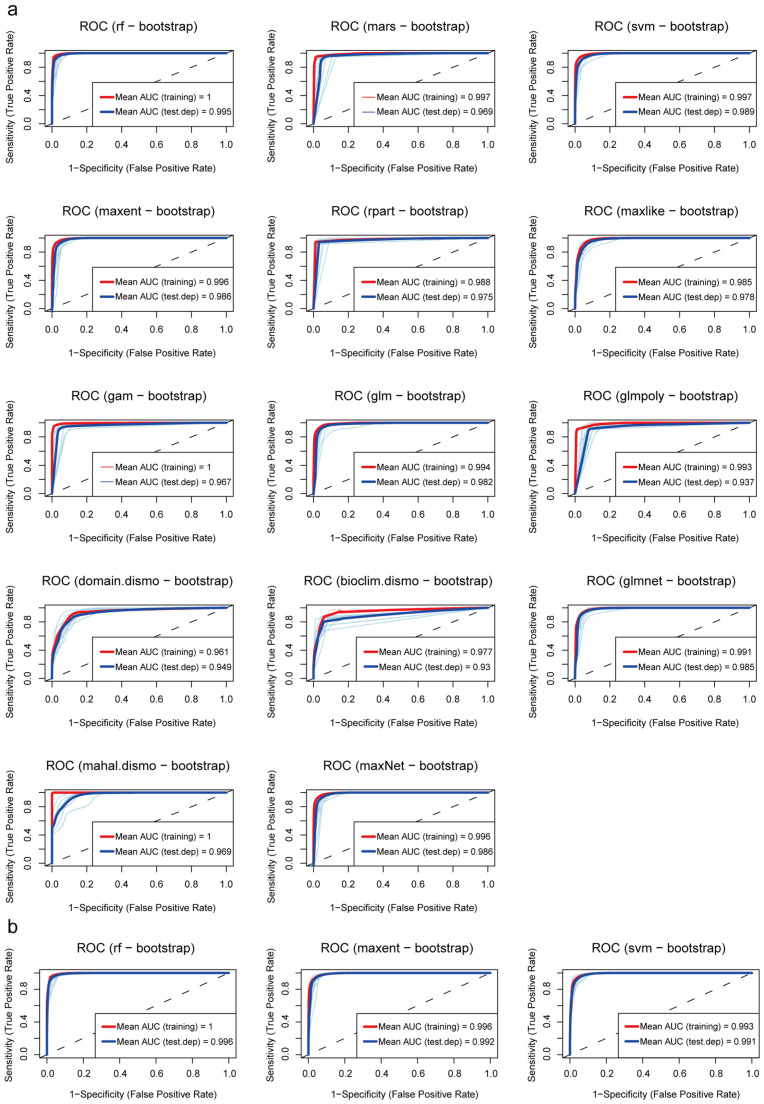
Receiver operating characteristic curves: (**a**) ROC curves of all fourteen models; (**b**) ROC curves of three ensemble models.

Firstly, the models were preliminarily screened based on the True Skill Statistic (TSS) to evaluate classification consistency (Table A1, Appendix A). When TSS > 0.95, five models —rf, mars, SVM, MaxEnt, and maxNet—exhibited high predictive consistency. Secondly, receiver operating characteristic (ROC) curve analysis was conducted (Figure 2a) to further assess overall predictive accuracy and model stability. When AUC (Training) > 0.99 and ΔAUC ≤ 0.01 (Table A1), models rf, svm, maxent, glmnet, and maxNet displayed high predictive accuracy with small differences between training and testing performance.

Based on the combined results of these two screening steps, we selected three models that simultaneously satisfied high classification consistency, predictive accuracy, and stability, namely random forests, Support Vector Machine, and MaxEnt, to construct the final ensemble model. The new model shows an average AUC of 0.996 and an average ΔAUC of 0.003, demonstrating its high reliability in predicting fall webworm distribution (Figure 2b).

To compare the influence of human activity and climatic factors on *H. cunea*-suitable habitats, we performed a contribution analysis of the influencing factors based on correlation measures. We then ranked them by importance, as shown in Table 1. The results indicate that human settlement density has the greatest impact on model performance, significantly exceeding other variables. This is followed by elevation, temperature seasonality (Bio3), annual precipitation (Bio12), and plantation forest cover. Among forest-related variables, plantation forest cover contributes more strongly to habitat suitability than natural forest cover. The relative contributions of other bioclimatic variables, such as natural forest cover and annual mean temperature, are relatively minor.

**Figure 3:**
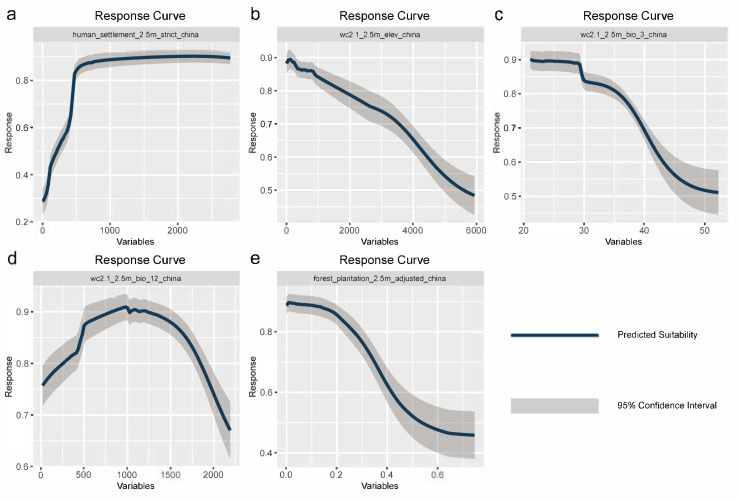
Response curves of high-contribution environmental factors on the distribution of fall webworm in China: (**a**) human settlement, (**b**) elevation, (**c**) bio3, (**d**) bio12, (**e**) forest plantation.

According to the response curves for each influencing factor generated by the model, the habitat suitability of *H. cunea* indicates a strong positive correlation with human settlement density. When the human settlement density index is below 200 (corresponding to rural and low-construction areas), the modeled suitability probability is relatively low, ranging from 0.25 to 0.5. When the settlement density index increases to around 500 (corresponding to county-level cities or urban outskirts), the habitat suitability probability rapidly rises above 0.8 and remains high (0.85–0.9) with further increases (Figure 3a). This trend indicates that the dispersal and establishment of *H. cunea* may be highly dependent on the migration corridors and habitats provided by human urban activities, such as urban green belts, street trees, transportation networks, and the movement of seedlings. Consistent with the variable importance analysis results (with human settlement density contribution > 50%), this confirms that human activities have a potentially significant impact on the habitat suitability distribution of *H. cunea*.

**Figure A2:**
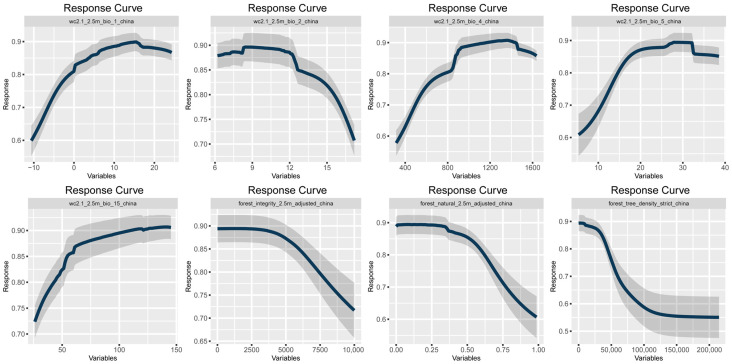
Response curves of the other eight environmental factors influencing the habitat suitability of fall webworm.

Apart from human activity factors, several natural ecological variables also exert a significant influence on the potential distribution of *H. cunea*. As elevation increases, the model-predicted suitability probability shows a continuous decline, remaining high (>0.85) in the 0–1000 m range, and then gradually declining (Figure 3b). Isothermality (BIO3), defined as the ratio of mean diurnal temperature range to annual temperature range, exhibits an overall negative relationship with habitat suitability (Figure 3c). When the proportion of diurnal temperature variation relative to annual temperature variation is below approximately 30%, the predicted suitability probability remains high (around 0.9). This pattern suggests that *H. cunea* is better adapted to temperate climates characterized by pronounced seasonality but relatively moderate diurnal temperature fluctuations. The effect of annual precipitation (BIO12) on suitability shows a typical unimodal pattern (Figure 3d); when precipitation is approximately 800–1200 mm, suitability probability reaches its highest (>0.9), whereas both too low and too high precipitation lead to decreased suitability, indicating that *H. cunea* forms stable populations more readily under moderate moisture conditions. The plantation forest cover variable shows a clear declining trend in habitat suitability as the proportion of plantation forest increases. When the proportion of plantation forest within an individual grid cell is low (approximately < 0.25), habitat suitability remains relatively high. However, as plantation forest cover increases further (>0.4), suitability declines markedly (Figure 3e). This result indicates that *H. cunea* is less adapted to densely planted, closed-canopy plantation forests and tends to prefer forest environments with more open structural characteristics. Response curve analyses further indicate that other influencing factors also affected the habitat suitability of *H. cunea* to varying degrees (Figure A2, Appendix A).

### 3.3. Current and Future Expansion of Fall Webworm’s Suitable Habitat

**Figure 4:**
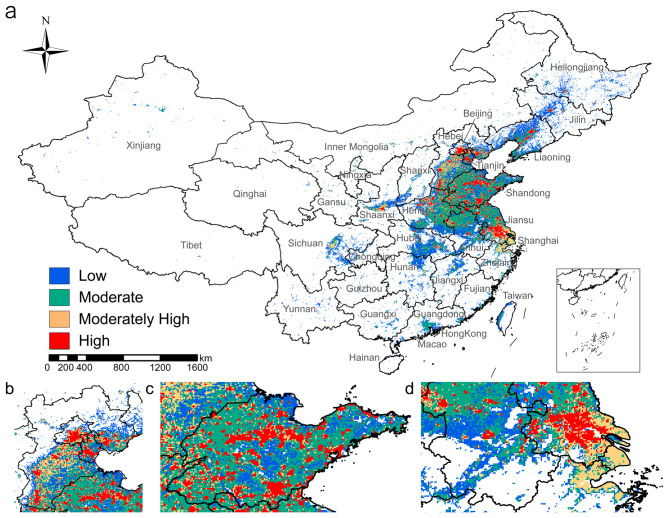
Distribution of potential suitable habitats for the fall webworm under current climate conditions: (**a**) national overview; (**b**) Beijing–Tianjin–Hebei central region; (**c**) Shandong Peninsula; (**d**) Yangtze River Delta.

**Figure A3:**
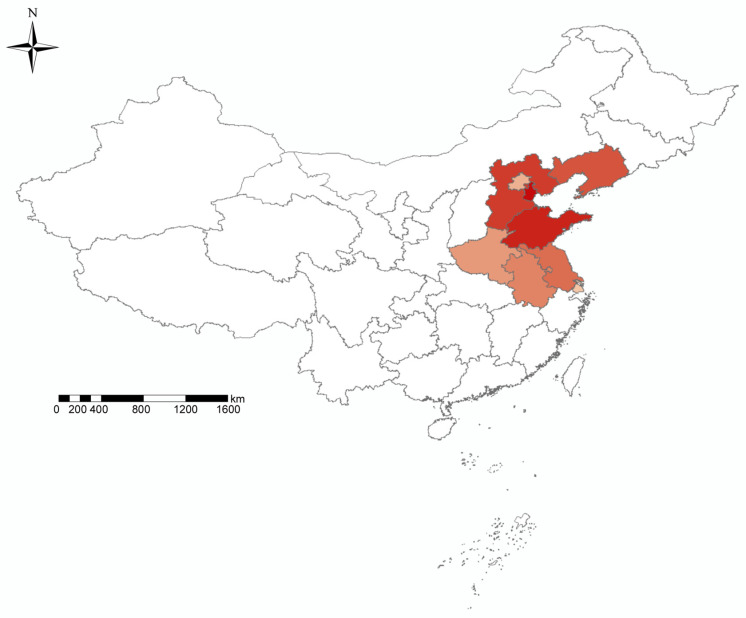
Current distribution of fall webworm in China and its correspondence with predicted suitable habitats under present climate conditions.

Building on previous analysis, we predicted the suitable habitat for *H. cunea* and found that, under current climate conditions, the potential suitable habitat in China exhibits a clear pattern of concentration in the east and sparsity in the west (Figure 4). There are three major hotspots: the Beijing–Tianjin–Hebei central region, the Shandong Peninsula, and the Yangtze River Delta (Figure 4b–d). These regions also correspond to the provinces where the damage caused by fall webworm is most severe. Moderately suitable areas form large, continuous regions that surround the highly suitable areas, including Liaoning, Hebei, Shandong, Henan, Jiangsu, and Anhui, and cover most of the currently known distribution areas (Figure 4 and Figure A3, Appendix A). The marginally suitable areas are mainly located in provinces where fall webworm has not been found, ranging from Jilin, Liaoning, and Inner Mongolia in the north to Xinjiang, Gansu, and Sichuan in the west; Guangdong, Guizhou, Jiangxi, Fujian, Hunan, and Hubei in the south; and Zhejiang and Taiwan in the east. Additionally, it is worth noting that in densely populated provinces such as Shaanxi, Sichuan, Guangdong, and Zhejiang, scattered areas of moderate to high suitability are still present (Figure 4a).

**Figure 5:**
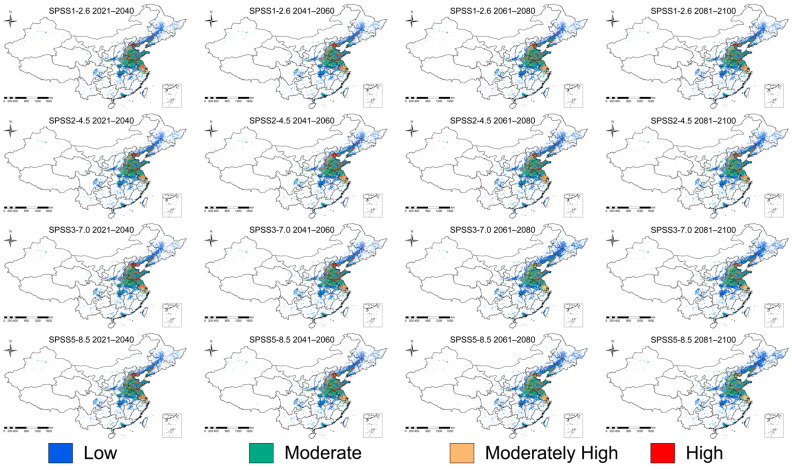
Distribution of potentially suitable habitats of fall webworm under different future climate scenarios.

**Figure 6:**
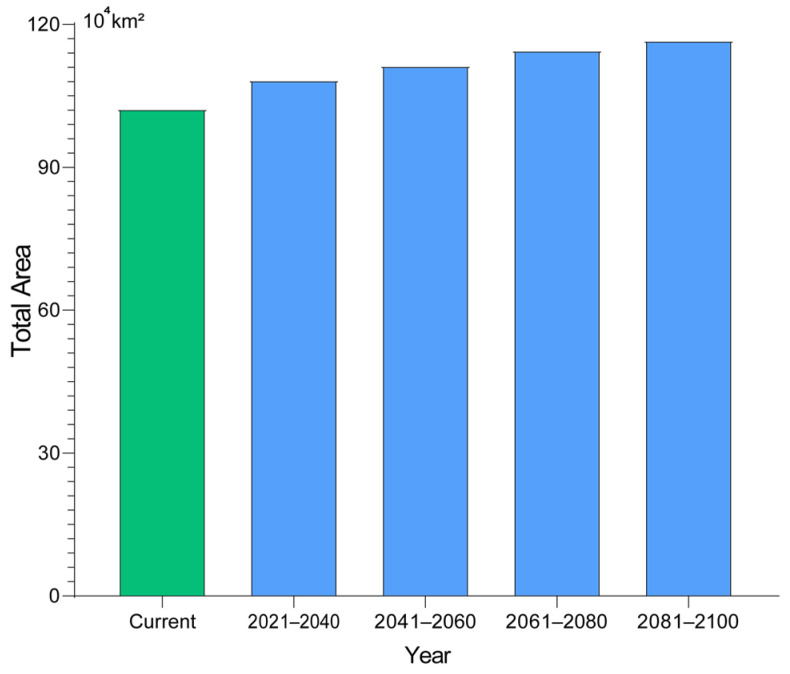
Comparison of the total suitable habitat area between the current period and the future projections under the SSP5-8.5 scenarios.

We examined potential changes in suitable habitat for the fall webworm under a range of climate scenarios. The results suggest that suitable areas may expand as climate change progresses, in particular under the high-emission scenario (SSP3-7.0 and SSP5-8.5). Model projections indicate that, by 2025, the area of total suitable habitat could approximate 102.33 × 10^4^ km^2^, with roughly 43.01 × 10^4^ km^2^ classified as low suitability, 35.42 × 10^4^ km^2^ as medium suitability, and about 23.90 × 10^4^ km^2^ as highly suitable. Under the low-emission scenario (SSP1-2.6), the expansion of suitable areas is relatively slow, with the total area increasing by 6.30 × 10^4^ km^2^ and 6.52 × 10^4^ km^2^ between 2041–2060 and 2061–2080, respectively, indicating a mild impact of climate change on its distribution. In contrast, under the medium emission scenario (SSP2-4.5), the suitable area grows faster, especially between 2061–2080, when it increases by 9.24 × 10^4^ km^2^ (9.03%). Under high-emission scenarios (SSP3-7.0 and SSP5-8.5), the growth is even more pronounced, particularly during 2081–2100, with suitable area increasing by 13.24 × 10^4^ km^2^ (12.94%) and 14.39 × 10^4^ km^2^ (14.07%). Overall, suitable habitat areas increase under all future SSP scenarios, with the greatest expansion occurring under the SSP5-8.5 scenario (Table 2, Figure 5 and Figure 6).

Based on the estimated suitable habitat areas and national average vegetation carbon density, we quantified the potential carbon stock exposed to fall webworm invasion under current and future climate scenarios. Vegetation carbon storage capacity was represented by the national average carbon density of China in 2025 (35.52 t C ha^−1^).

Under current climate conditions, the total suitable area was estimated at 102.33 × 10^4^ km^2^, corresponding to a potential exposed carbon stock of approximately 3634.8 TgC. Under the SSP5–8.5 scenario for the late 21st century (2081–2100), the suitable habitat area expanded to 116.72 × 10^4^ km^2^, resulting in an estimated exposed carbon stock of approximately 4145.9 TgC. Compared with current conditions, this represents an increase of 14.39 × 10^4^ km^2^ in suitable habitat area, equivalent to an additional 511.1 TgC of carbon stock potentially exposed to invasion risk.

It should be emphasized that the above estimates are based on several simplifying assumptions, including spatially uniform vegetation carbon density and equal potential exposure risk across all suitable habitats. Accordingly, this analysis is intended to provide a first-order, exposure-based assessment, characterizing the spatial overlap between the potential invasion range of fall webworm and national-scale vegetation carbon stocks, rather than a quantitative prediction of actual vegetation carbon loss.

## 4. Discussion

In the current Anthropocene era, the spread of invasive species is influenced not only by climate conditions but also by human activities [31,32]. As global biological invasions become increasingly human-facilitated, high-density cities, serving as hubs of human settlement and trade, are now the major hotspots and corridors for the establishment and spread of alien species. Hence, a comprehensive understanding of areas prone to potential spread is essential for the development and implementation of proactive control measures [31]. Our results suggest that, in the case of *H. cunea*, human settlement density emerges as the strongest correlate of predicted habitat suitability, appearing to exceed the contribution of traditional forest structural or climatic variables. This is supported by the response curve from our ensemble model, which remains above 0.8 at settlement densities greater than 500. However, this pattern likely reflects a combination of factors associated with human presence, including urban greenbelts, roadside vegetation, nursery and plant trade, and transportation networks, which collectively enhance dispersal opportunities and reporting likelihood [33].

Natural environmental factors also exert an important regulatory influence on the habitat suitability of *H. cunea*. Our study indicates that *H. cunea* tends to favor warm, humid, climatically stable, and low-lying regions, with suitability markedly increasing under conditions of 800–1200 mm of annual precipitation, intermediate isothermality, and elevations below 1000 m. This ecological preference may help explain the species’ extensive distribution across regions such as China’s eastern coastal zone and the North China Plain [34]. The combination of favorable climatic conditions, abundant agricultural and forest vegetation, and intensive human activity in these regions may collectively contribute to their heightened vulnerability to the further expansion of *H. cunea*. In contrast, a decline in habitat suitability is observed in areas with extensive plantation forest cover, a trend that may reflect reduced light availability within denser stands. This pattern, in turn, suggests that *H. cunea* may be more prone to occupy and disperse through relatively open forest areas where shading is limited.

Future climate change may contribute to an increase in the extent of highly suitable habitat for *H. cunea*. Projections across the four SSP scenarios indicate the potential for a gradual expansion of suitable areas from 2021 to 2100, particularly under the high-emission scenario (SSP5-8.5), which is estimated to ultimately reach approximately 116.72 × 10^4^ km^2^ by the end of the 21st century. The anticipated expansion of *H. cunea* also implies broader ecosystem-level consequences. By integrating suitable habitat projections with vegetation carbon density, our results highlight that a substantial amount of ecosystem carbon stock is currently located within areas potentially exposed to invasion, and that this exposure may further increase under high-emission climate scenarios. Again, we reiterate that this estimate does not seek to represent actual carbon losses but rather serves as an upper-bound assessment of the carbon stocks that may be placed at risk as invasion fronts advance. Similar first-order, exposure-based assessments are conventionally used in large-scale evaluations of carbon sink vulnerability, where the spatial overlap between disturbance risk and carbon storage capacity is applied to contextualize potential ecosystem impacts [1,30]. Despite the inevitable omission of fine-scale heterogeneity in vegetation and management, this approach provides an intuitive metric for linking climate-driven invasion dynamics with ecosystem service risks.

This study adopted an ensemble model built from a selective combination of Random Forest, MaxEnt, and Support Vector Machine (SVM), integrating multidimensional factors such as geography, climate, human activity, and vegetation cover. Effective improvements in the accuracy and reliability of predictions were made possible by such a multi-model approach, arguably overcoming the limitations of single-model methods. Early predicational studies primarily used single-model methods individually, such as MaxEnt, GARP, or CLIMEX, along with limited distribution records from China. These studies generally indicated that northeastern, northern, and eastern China have high habitat suitability for fall webworm [35,36]. Later studies gradually incorporated future climate change scenarios, emphasizing that global warming could further expand the suitable habitat for fall webworm [37]. In recent years, more studies have focused on niche shifts between native and invaded areas, highlighting climate-limiting factors, particularly the role of precipitation seasonality, and drawing attention to the point that models based solely on native range data can severely underestimate the invasion risk in China [34]. This study provides scientific evidence for monitoring and controlling fall webworm across China. The results suggest that priority should be given to monitoring areas already invaded by *H. cunea* and those that are highly suitable, especially in regions with dense human activity, providing data informing precise invasive species management strategies.

## Figures and Tables

**Table 1:** Contribution of environmental variables to the model predicting the distribution of the fall webworm.

Environmental Variable	Contribution (%)
Human settlement	51.8
Elevation	4
Isothermality (Bio3)	2.2
Annual precipitation (Bio12)	2.1
Forest plantation	2
Forest natural	1.7
Annual mean temperature (Bio1)	1.5
Forest integrity	1.4
Mean diurnal temperature range (Bio2)	1.3
Forest tree density	1.3
Max. temperature of warmest month (Bio5)	1.3
Temperature seasonality (Bio4)	1.2
Precipitation seasonality (Bio15)	1

**Table 2:** Area suitable for fall webworm in different periods and scenarios.

Climate Scenario	Period (Year)	Low Suitable Area (10^4^ km^2^)	Medium Suitable Area (10^4^ km^2^)	High Suitable Area (10^4^ km^2^)	Total Area (10^4^ km^2^)	Area Change (10^4^ km^2^)	Area Change Ratio (%)
Current	2025	43.01	35.42	23.90	102.33	-	-
SSP1-2.6	2021–2040	45.85	37.66	24.59	108.10	5.77	5.64
	2041–2060	46.91	37.31	24.40	108.63	6.30	6.16
	2061–2080	47.02	37.48	24.34	108.85	6.52	6.38
	2081–2100	47.92	37.15	24.29	109.37	7.04	6.88
SSP2-4.5	2021–2040	45.78	38.40	24.64	108.81	6.49	6.34
	2041–2060	47.46	37.95	24.59	110.00	7.67	7.50
	2061–2080	49.14	38.00	24.42	111.57	9.24	9.03
	2081–2100	49.25	37.29	24.01	110.55	8.23	8.04
SSP3-7.0	2021–2040	45.07	39.16	25.01	109.24	6.91	6.75
	2041–2060	46.98	38.85	24.85	110.68	8.36	8.17
	2061–2080	57.27	36.25	23.20	116.72	14.39	14.07
	2081–2100	53.89	37.64	24.04	115.56	13.24	12.94
SSP5-8.5	2021–2040	46.10	37.74	24.55	108.39	6.06	5.92
	2041–2060	48.39	38.36	24.62	111.38	9.05	8.85
	2061–2080	52.63	37.95	24.03	114.61	12.29	12.01
	2081–2100	57.27	36.25	23.20	116.72	14.39	14.07

## Data Availability

The original contributions presented in this study are included in the article/Supplementary Material. Further inquiries can be directed to the corresponding authors.

## Supplementary Materials

The following supporting information can be downloaded at: https://www.mdpi.com/article/10.3390/insects17020154/s1, Table S1: *Hyphantria_cunea* DistributionDataSource.

## Appendix A

**Table A1:** Performance statistics of different species distribution modeling algorithms for *H. cunea*.

Methods	TSS	AUC (Training)	AUC (Test.Dep)	ΔAUC
rf	0.97	1	0.995	0.005
mars	0.95	0.997	0.969	0.028
svm	0.95	0.997	0.989	0.008
maxent	0.95	0.996	0.986	0.010
rpart	0.94	0.988	0.975	0.013
maxlike	0.9	0.985	0.978	0.007
gam	0.93	1	0.967	0.033
glm	0.9	0.994	0.982	0.012
glmpoly	0.91	0.993	0.937	0.056
domain.dismo	0.86	0.961	0.949	0.012
bioclim.dismo	0.9	0.977	0.930	0.047
glmnet	0.87	0.991	0.985	0.006
mahal.dismo	0.92	1	0.969	0.031
maxNet	0.95	0.996	0.986	0.010
